# Deep Versus Superficial Dry Needling for Neck Pain: A Systematic Review of Randomised Clinical Trials

**DOI:** 10.3390/medicina61101832

**Published:** 2025-10-13

**Authors:** Anas M. Alhakami, Ahmad Sahely, Ali M. Y. Alshami

**Affiliations:** 1 Department of Physical Therapy, College of Applied Medical Sciences, Imam Abdulrahman Bin Faisal University, Dammam 31441, Saudi Arabia; alshami@iau.edu.sa; 2 Department of Physical Therapy, College of Nursing and Health Sciences, Jazan University, Jazan 45142, Saudi Arabia; asahely@jazanu.edu.sa

**Keywords:** dry needling, neck pain, pain management, physical therapy, systematic review

## Abstract

*Background and Objectives*: Research examining the difference between the effects of deep and superficial dry needling on myofascial trigger points (MTrPs) in the upper trapezius muscle is limited. Thus, this systematic review was conducted to compare the effects of these two dry needling techniques on pain and functional disability in adults with neck pain who demonstrated MTrPs. *Materials and Methods*: Randomised clinical trials (RCTs) were identified through an electronic search in PubMed, Scopus, Web of Science, Embase, Google Scholar, Dimensions and OpenAlex from inception until 22 September 2024. Only English-language studies were considered. Best-evidence synthesis was utilised to interpret the results of the included RCTs. *Results:* Of the 192 records obtained, 8 RCTs were included (2 with a low risk of bias, 4 with some risk-of-bias concerns and 2 with a high risk of bias). Overall, both deep and superficial dry needling provided short-term alleviation of pain and functional disability. No clinically meaningful differences were found between the two dry needling techniques. *Conclusions:* Deep and superficial dry needling seem to have similar positive effects on pain and functional disability in patients with neck pain exhibiting MTrPs.

## 1. Introduction

The human musculoskeletal system, the largest system in the human body, is prone to various types of pain [1]. Musculoskeletal pain affects around 80% of the population [2]. Myofascial pain syndrome (MPS) is a common musculoskeletal disorder, with lifetime prevalence estimates ranging from 30% to 85% depending on the population studied. In clinical settings, approximately 30% of primary care patients and up to 85% of pain clinic attendees are affected [3,4]. In Saudi Arabia, approximately 69% of the population experience symptoms of MPS at some points in their lives [5].

The most widely accepted definition of MPS, established by Simons and Travell in 1983, is regional pain linked to myofascial trigger points (MTrPs) [6]. MTrPs are commonly found in the upper back, with 38–65% located in the trapezius muscle [7]. These MTrPs are postulated to arise from trauma or overuse, leading to local energy crises and motor endplate dysfunction, which result in continuous muscle contraction and local hypoxia [8]. MTrPs exhibit distinctive characteristics, such as tender points, palpable taut bands, referred pain, local twitch response and/or restricted range of motion [9].

Physiotherapy for myofascial trigger point syndrome (MTrPS) includes non-invasive approaches such as manual therapy and therapeutic exercises [10,11], as well as invasive techniques like dry needling [11,12]. While all have been shown to reduce pain and improve function, they differ in their mechanisms of action and the potential risks associated with treatment. Dry needling was developed by Karel Lewit in 1979 [13] and has two primary forms in terms of depth: superficial and deep [14]. Superficial dry needling involves inserting the needle into the skin at a depth of 5–10 mm without making it reach the MTrPs, whereas deep dry needling involves deep skin penetration into the muscle to target the MTrPs [3,15]. Each of these dry needling techniques has a different suggested mechanism for its effect on MTrPs [15,16].

Several clinical studies have compared the effectiveness of deep dry needling with that of superficial dry needling in patients with neck pain [17,18,19,20]. These studies obtained contradictory results. A systematic review with a meta-analysis by Griswold et al. (2019) compared the effectiveness of deep dry needling with that of superficial dry needling or acupuncture in treating painful spinal disorders [15]. The review includes 12 quasi-experimental and randomised clinical trials (RCTs). Of these, only two studies [19,20] compared the effectiveness of deep dry needling with that of superficial dry needling on upper-trapezius MTrPs, and their results were contradictory [16]. To our knowledge, the systematic review by Griswold et al. (2019) is the only one that compared the effectiveness of deep dry needling with that of superficial dry needling in patients with MTrPs up to that time [15]. Since then, several new RCTs have been published. Notably, Hernández-Secorún et al. (2023) [21] conducted a systematic review including 15 RCTs and reported that both deep and superficial dry needling provided short-term pain relief and functional improvement in patients with neck pain and MTrPs, with no clinically meaningful differences between the two techniques. Similarly, Rodríguez-Huguet et al. (2022) [22] compared the immediate and delayed effects of superficial and deep dry needling on pain and disability in patients with active upper trapezius MTrPs, also reporting comparable outcomes.

Thus, the current systematic review was conducted to compare the effects of deep and superficial dry needling on pain and functional disability reduction in adults with neck pain demonstrating MTrPs.

## 2. Materials and Methods

### 2.1. Protocol Registration

This review was prospectively registered in the International Prospective Register of Systematic Reviews (PROSPERO) on 19 September 2024 (CRD42024587994). It followed the Preferred Reporting Items for Systematic Reviews and Meta-Analysis (PRISMA) guidelines [23].

### 2.2. Eligibility Criteria

RCTs involving adult patients with neck pain and upper-trapezius MTrPs that compared the effects of deep and superficial dry needling and used pain intensity and functional disability as primary outcome measures were included in the current systematic review. Only studies published in the English language were considered. Studies that included individuals with radiculopathy, whiplash injury or post-surgery or who used analgesic medications or had been treated with other physiotherapy interventions were excluded. The inclusion and exclusion criteria are presented in Table 1 according to the PICO framework [24].

### 2.3. Search Strategy

The search strategy was developed by a health sciences librarian and reviewed by the primary investigator A.M.A. Two reviewers A.M.A and A.S simultaneously searched the PubMed, Scopus, Web of Science, Embase, Google Scholar, Dimensions and OpenAlex databases from inception to 22 September 2024. The keywords included ‘neck pain’, ‘cervical pain’, ‘superficial dry needling’, ‘deep dry needling’, ‘trigger points’ and ‘MPS’. The Boolean ‘AND’ operator was employed to combine subjects, while the ‘OR’ operator was used to link headers. The Peer Review of Electronic Search Strategies (PRESS) checklist was applied to improve the search strategy [25]. Based on its recommendations, adjustments were made to ensure consistent use of Boolean operators, removal of redundant terms, refinement of truncation and phrase searching, and inclusion of grey literature sources (Google Scholar, Dimensions, and OpenAlex). These modifications enhanced both the sensitivity and specificity of the search. The reference lists of studies were also searched manually for additional relevant studies. Details of the search strategy are attached in Appendix A.

### 2.4. Study Selection

The studies identified during the search were imported into the Zotero 7.0.7 reference manager software (Roy Rosenzweig Center for History and New Media, George Mason University, Fairfax, VA, USA). After eliminating duplicates using Zotero’s ‘Duplicate Items’ and manual review in the Excel sheet, A.M.A and A.S screened the titles and abstracts for eligibility. They then independently screened the full-text articles. If there were discrepancies between the results obtained by the two reviewers, a third reviewer (A.M.Y.A) was consulted.

### 2.5. Risk-of-Bias Assessment

The risk of bias of the selected studies was evaluated using the Cochrane Risk of Bias 2 (RoB 2) tool for randomised trials, which included five domains: randomisation process, deviation from intended interventions, missing outcome data, outcome measurement and selection of reported results. Studies were classified as either having a low risk of bias (1), having some risk-of-bias concerns (2) or having a high risk of bias (3) according to the established criteria [26].

### 2.6. Data Extraction and Synthesis

A.M.Y.A and AS independently extracted the following data from each eligible study: author(s), year of publication, participants’ characteristics, diagnostic criteria, interventions, number of sessions, follow-ups, outcome measures and main findings.

Evidence synthesis was performed according to the best-evidence synthesis principles outlined by Slavin, 1995 [27]. Owing to the clinical variability among the included studies in needle insertion depth, session frequency, follow-up lengths and outcome measurement tools, quantitative synthesis of data through meta-analysis was impracticable. The results were summarised descriptively, emphasising the identification of trends and patterns regarding the efficacy of deep and superficial dry needling in patients with neck pain. Increased emphasis was placed on studies with a low risk of bias and a more rigorous methodology. The synthesis emphasised the clinical importance of the interventions when applicable, using both qualitative patterns and quantitative results from high-quality studies.

### 2.7. Statistical Analysis

The Statistical Package for the Social Sciences software (SPSS) (version 29.0, IBM Corporation, Armonk, NY, USA) was used for data analysis. The inter-rater agreements between the reviewers for article screening and risk-of-bias assessment were calculated using Cohen’s kappa coefficient (κ) and the percentage of agreement, respectively. The Cohen’s kappa coefficient values are classified as no (≤0), none-to-little (0.01–0.20), reasonable (0.21–0.40), moderate (0.41–0.60), substantial (0.61–0.80) and almost perfect agreement (0.81–1.00). A 95% confidence interval (CI) for κ was established to evaluate the accuracy of the agreement estimate. Mean differences (MD’s) and 95% CIs were reported, if applicable, to estimate the improvements in outcome measures.

## 3. Results

### 3.1. Study Selection

**Figure 1:**
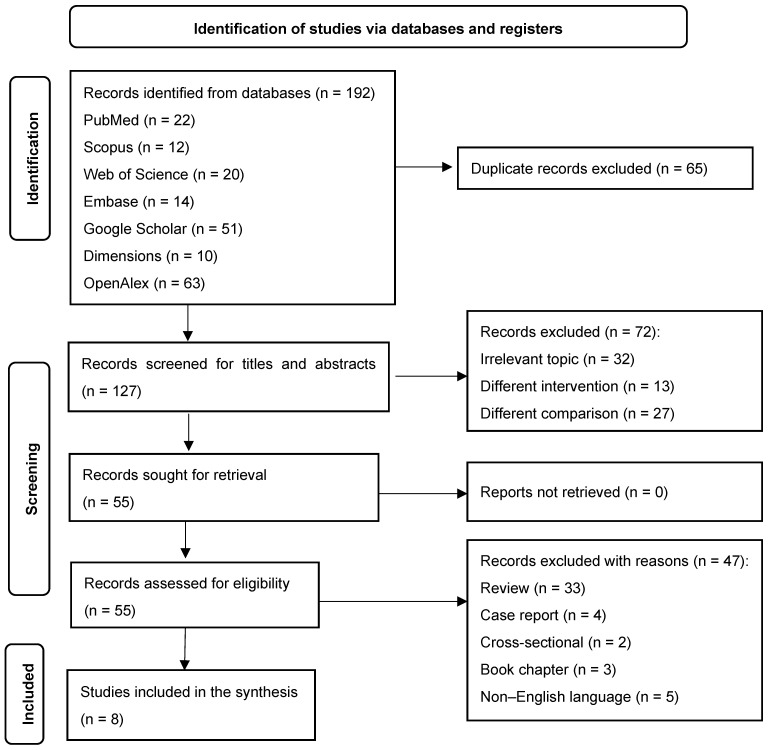
Flow diagram of the identified studies in the current systematic review.

Of the 192 identified records, 127 were screened (Figure 1). Eight studies were critically appraised in this review. Of these studies, seven [17,18,20,28,29,30,31] were identified via databases, and one [19] was identified via manual research. The inter-rater agreement for screening was perfect (κ = 1.00; 95% CI 1.00, 1.00; Table 2). There were no discrepancies between the two reviewers, indicating that there were no instances in which one reviewer included an article while the other excluded it. The percentage of agreement for screening the title and abstract was 100% (8/8 studies).

### 3.2. Study Characteristics

Table 3 summarises the data extracted from the eight studies included in the current systematic review [17,18,19,20,28,29,30,31]. A total of 525 participants were recruited. All studies compared the effects of deep and superficial dry needling. Six of the studies measured both pain intensity and functional disability [17,18,20,29,30,31] and two studies measured only pain intensity using outcome measures such as the visual analogue scale (VAS) and conditional pain modulation (CPM) [19,28]. Pressure pain threshold (PPT) was measured in three studies [28,30,31], four-point pain intensity rating scale in one study [20], self-reported pain (NRS-101) in one study [30], conditioned pain modulation (CPM) in one study [28], and headache index (HI) and trigger point tenderness in one study [20]. The Neck Disability Index (NDI) was measuered in three studdies [17,18,29] and Functional Rating Index (FRI) in one study [20]. Other outcome measures including cervical range of motion (CROM) that was measured in four studies [17,20,29,31], maximal isometric muscle force (Fmax) and rate of force development (RFD) in one study [30], surface electromyography (sEMG) in one study [18], cervical motor control (CMC) in one study [29] and ultrasonic evaluation in one study [19]. Thus, the results of these studies could not be pooled in a meta-analysis due to heterogeneity.

### 3.3. Risk of Bias

Two of the eight RCTs [28,29] had a low risk of bias. Four RCTs [18,19,30,31] were rated as having some risk-of-bias concerns because of a lack of randomisation or selection of reported outcomes. Two trials [17,20] had a high risk of bias due to a lack of randomisation, bias due to deviation from intended treatments and bias in outcome measurement. A common strength across all eight RCTs was the low risk of bias due to missing outcome data and outcome measurements. The κ and percentage of agreement for risk of bias between the reviewers were moderate (0.78 and 75%, respectively; Table 4). However, six RCTs [17,18,19,20,30,31] had either some risk-of-bias concerns or a high risk-ofbias due to randomisation (Table 5).

### 3.4. Intervention Protocol

Variability was observed in both the deep and superficial dry needling techniques in the trials. For deep dry needling, Sedighi et al., 2017 [20] described the procedure only as deep needling for trigger points and kept the needle inserted into the skin for 15 min before removal. Ezzati et al., 2018 [17] and Sarrafzadeh et al., 2018 [19] used 50 mm-long and 0.25 mm diameter needles, inserting them into the skin 8 times, rapidly moving them back and forth without making them exit the skin and then keeping them in situ for 5 min. Chys et al., 2023 [28], Hoseininejad et al., 2023 [18] and Navarro et al., 2022 [31] used 40 mm long and 0.2- to 0.25 mm diameter needles and performed 8–10 in-and-out movements. Martín-Rodríguez et al., 2019 [29] used 25 mm × 0.25 mm needles for 8–10 in-and-out movements, whereas Myburgh et al., 2012 [30] used a 25 mm long needle and repeatedly inserted it into the skin and rotated it clockwise for 90 s. To sum up, for deep dry needling, a 0.2- to 0.25 mm diameter needle was used, which was inserted into the skin up to a depth of 25–50 mm for 1.5–15 min and either kept inserted during the entire session or inserted and removed 8–10 times.

For superficial needling, Navarro et al., 2022 [31] described subcutaneous needle insertion and performed three needle rotations with 3 min intervals. Sedighi et al., 2017 [20] inserted the needle subcutaneously and kept it in place for 15 min before removal. Ezzati et al., 2018 [17] and Sarrafzadeh et al., 2018 [19] inserted a 0.25 mm diameter needle into the skin up to a depth of 5 mm (distance of the needle from the plastic tube) and rapidly moved it back and forth 8 times, without making it exit the skin, and then kept it in situ for 5 min. Myburgh et al., 2012 [30] repeatedly inserted a 5- to 10 mm long and 0.25 mm diameter needle into the skin superficially and rotated it clockwise for 90 s. Hoseininejad et al., 2023 [18] inserted a 0.2 mm diameter needle into the skin up to a depth of 13 mm and kept it in place for 2–3 min. Chys et al., 2023 [28] inserted the needle subcutaneously and performed in-and-out movements 10 times. Martín-Rodríguez et al., 2019 [29] employed a superficial needle insertion technique using a 0.25 mm diameter needle at a depth of 15 mm, swiftly moving the needle in and out 10 times. In summary, superficial dry needling using a 0.2- to 0.25 mm diameter needle was performed up to a depth of 5–15 mm for 1.5–15 min, either keeping the needle underneath the skin for the entire session or inserting and removing it 10 times.

### 3.5. Intervention Duration

Six of the eight studies applied only one session of deep or superficial dry needling, with variations in follow-up duration: immediately [28], 1 day [30], 1 week [18,20,31] and 1 month [29]. Only two studies [17,19] applied three sessions of deep or superficial dry needling for 2 weeks.

### 3.6. Effect on Pain Severity

**Figure 2:**
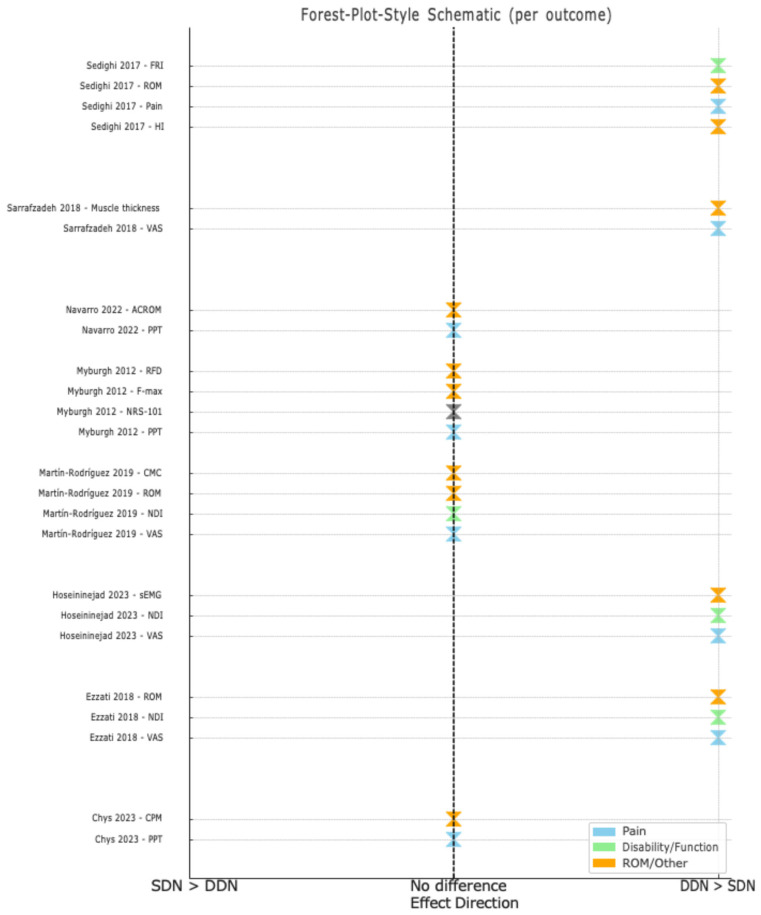
Forest-plot-style summary of the comparative effects of deep and superficial dry needling on neck pain and functional outcomes from the reviewed studies [17,18,19,20,28,29,30,31].

Two RCTs [17,18] found that pain intensity as measured by VAS was reduced significantly after both deep (MD = 2.2/10 and 2.64/10) and superficial dry needling (MD = 1.8/10 and 2.36/10), without significant differences between the two treatments (*p* > 0.05). In Sarrafzadeh et al., 2018 [19] study, deep dry needling resulted in a greater decrease in VAS (MD = 3.98/10, 95% CI = 3.58–4.38) compared to superficial dry needling (MD = 2.0/10, 95% CI = 1.65–2.35). In Martín-Rodríguez et al., 2019 [29] study, superficial dry needling resulted in a higher reduction in VAS (MD = 2.2/10) compared to deep dry needling (MD = 0.8/10). In their trials, Myburgh et al., 2012 [30] and Navarro et al., 2022 [31] used PPT to measure pain intensity and found that deep dry needling significantly reduced PPT (MD = ‒33.99 kPa and 0.43 kg/cm^2^) compared to superficial dry needling (MD = 3.49 kPa and 0.12 kg/cm^2^). In another trial by Chys et al., 2023 [28], both deep and superficial dry needling improved PPT (MD = 1.03 N/cm^2^ and 1.15 N/cm^2^, respectively), with no significant differences between the two techniques (*p* > 0.05). Myburgh et al., 2012 [30] used NRS-101 and observed a higher reduction in deep dry needling (MD = 2.18 points, 95% CI = 1.07–3.29) than in superficial dry needling (MD = 0.80 points, 95% CI = 0.48–1.12). Chys et al., 2023 [28] found improvement in the relative CPM (at the quadriceps) in deep dry needling (MD = 13.52%, 95% CI = 0.46–26.59), but not in superficial dry needling (MD = 1.81%, 95% CI = ‒10.62–14.23), although there was no improvement in the absolute CPM (at the trapezius muscle) in both dry needling approaches (*p* > 0.05). Sedighi et al., 2017 [20] used trigger point tenderness and HI and observed reductions after deep dry needling (trigger point tenderness: MD = 1.49°, 95% CI = 0.94–2.04; HI: MD = 8.07, 95% CI = 5.18–10.96) and superficial dry needling (trigger point tenderness: MD = 1.29°, 95% CI = 0.72–1.86; HI: MD = 8.20, 95% CI = 5.49–10.91), with no differences between the two techniques (*p* > 0.05) (Figure 2).

### 3.7. Effect on Functional Disability

In two RCTs [18,29], functional disability was alleviated, as demonstrated by NDI, in both deep dry needling (MD = 5.2 points and 6.4 points) and superficial dry needling (MD = 7.2 points and 5.18 points). In another trial [17], however, there was greater improvement in NDI with deep dry needling (MD = 8 points) than with superficial dry needling (MD = 2 points). Sedighi et al., 2017 [20] used FRI and reported that both deep and superficial dry needling improved function, but higher improvement was observed in the deep dry needling group (MD = 28.7%, 95% CI = 20.43–36.89) than in the superficial dry needling group (MD = 16.4%, 95% CI = 10.86–21.94) (Figure 2). 

### 3.8. Effects on Other Outcome Measures

Two RCTs [20,31] evaluated CROM and found that it improved in both deep and superficial dry needling, but higher improvement was observed in the deep dry needling group. However, in both studies, the exact MD and 95% CI were not provided. Myburgh et al., 2012 [30] used RFD and Fmax in their trial and found that both deep and superficial dry needling did not affect these outcomes (*p* > 0.05). Hoseininejad et al., 2023 [18] used sEMG and found that deep dry needling increased muscle activity (MD = 9.88 root mean square [RMS]) compared to superficial dry needling (MD = 1.04 RMS). Martín-Rodríguez et al., 2019 [29] measured CMC by testing the cervical joint position error and found that both deep and superficial dry needling improved CMC (MD = 2.1 cm and 1.8 cm, respectively), with no statistically significant differences between the two techniques (*p* > 0.05). Sarrafzadeh et al., 2018 [19] used diagnostic ultrasound to measure the upper-trapezius-muscle thickness at rest, fair and normal contraction and reported that both deep and superficial dry needling enhanced muscle thickness (rest MD = 0.55 mm and 0.10 mm, respectively; fair MD = 0.49 mm and 0.12 mm; normal MD = 0.59 mm and 0.09 mm), with no significant differences between the two techniques (*p* > 0.05) (Figure 2). 

## 4. Discussion

The purpose of the current systematic review was to evaluate the effectiveness of superficial and deep dry needling of MTrPs in reducing pain intensity and improving functional disability in patients with neck pain. MTrP formation occurs when a muscle is overloaded or subjected to repetitive strain. Sustained contraction can lead to inadequate blood flow (ischemia) in the muscle fibres, creating an ‘energy crisis’, where the muscle tissue cannot receive enough oxygen and nutrients to meet its metabolic demands. This energy crisis triggers the excessive release of acetylcholine at the neuromuscular junction, resulting in continuous muscle contraction [6,8]. The sustained contraction further perpetuates the cycle by compressing blood vessels, worsening ischemia and leading to the accumulation of metabolic by-products, such as lactic acid. Ultimately, this ongoing cycle results in localised pain, muscle stiffness and the formation of a myofascial trigger point [6,8]. This is the most widely accepted explanation of MTrP formation because it has been supported by evidence from several studies using different diagnostic modalities, such as ultrasonography, EMG and micro dialysis [4,8,32,33]. However, it is important to note that the pathophysiology of MTrPs has not been unequivocally confirmed, and several competing theories have been proposed. For example, the central sensitisation hypothesis suggests that trigger points may reflect altered nociceptive processing within the central nervous system, while the neuromotor dysfunction theory attributes MTrP development to impaired motor control and altered muscle activation patterns [16]. Thus, while the energy crisis hypothesis provides a useful framework, the exact mechanism underlying MTrP formation remains a subject of debate.

The mechanism behind the effects of deep dry needling is believed to be related to its ability to reach the MTrP, where it reduces end plate noise [16,34]. Additionally, the observed decrease in acetylcholine levels may lead to increased muscle blood flow and oxygenation and reduced sarcomere contracture [16,34]. Thus, the key factor in using deep dry needling is the mechanical stimulation of the trigger point. This stimulation leads to improvements in fibre structure, reductions in local stiffness, enhanced blood circulation, production of muscle actin and enhancement of the repair of fascia in damaged areas [35].

The mechanism of action of superficial dry needling is postulated as stimulating Aδ nerve fibres, with the consequent release of opioid peptides from enkephalinergic inhibitory interneurons in the dorsal horn. These peptides then inhibit the intradorsal horn transmission of nociceptive information conveyed to the spinal cord via group IV sensory afferents from the MTrP [3]. Thus, in superficial dry needling, mechanical stimulation is minimal and limited to just beneath the skin’s surface, not reaching the MTrP. In addition to this neurophysiological pathway, other mechanisms have also been proposed. Superficial dry needling may modulate sympathetic nervous system activity, leading to changes in autonomic regulation of pain perception, and may induce local improvements in tissue perfusion and oxygenation, thereby contributing to pain relief and muscle relaxation [36]. These additional mechanisms suggest that the effects of superficial dry needling may extend beyond the activation of descending inhibitory systems, although its overall effectiveness remains debated [36,37].

In addition to these neurophysiological and physiological mechanisms, psychosocial factors may also influence the outcomes of dry needling. Patient expectations and therapist communication strategies can shape pain perception and post-needling soreness. For instance, an experimental trial demonstrated that verbal suggestion significantly altered post-needling pain responses and pain modulation, underscoring the importance of patient–therapist interaction in treatment outcomes [38]. This highlights that the effectiveness of dry needling cannot be attributed solely to the mechanical or neurophysiological effects of needle insertion, but must also be understood within the broader biopsychosocial framework of pain management.

Our review included eight RCTs with a risk of bias ranging between low [28,29], with some concerns [18,19,30,31] and high [17,20]. Due to the nature of the intervention, blinding of the therapist was not feasible in the included studies, potentially introducing bias into the results. Overall, the synthesised evidence suggests that both the superficial and deep dry needling approaches may be effective in providing short-term reductions in pain associated with MTrPs in patients with neck pain. Most of the RCTs in the current review demonstrated no difference in pain effect between deep and superficial dry needling. Our findings are similar to those of previous RCTs on other body parts, such as the lumbar spine [39] and knee [40], which found no significant differences between the two dry needling approaches. However, two RCTs with some risk-of-bias concerns [19,30] demonstrated a statistically significant pain reduction with deep dry needling compared to superficial dry needling. Notably, the reported pain reductions in these studies (1.4 points for NRS-101 and 2.0 points for VAS) exceeded the established minimal clinically important differences (MCIDs) for neck pain (1.3 for NRS-101 and 0.8 for VAS) [41,42], indicating potential clinical relevance. While these findings highlight the potential short-term benefits of dry needling for pain and disability, evidence regarding medium- and long-term effectiveness remains scarce. Only a small number of included studies extended follow-up beyond the immediate post-treatment period, and few examined outcomes beyond 3 months. This limitation is of particular importance in the management of chronic neck pain, where sustained improvements are essential. The lack of robust medium- and long-term data reduces the certainty of our conclusions and underscores the need for future trials with extended follow-up periods to determine whether the observed benefits are maintained over time.

A previous systematic review examining the effects of dry needling (superficial and deep) on pain and functional disability in people with spinal pain indicated that deep dry needling was statistically superior to superficial dry needling in reducing pain, although this difference may not be clinically meaningful [15]. On the other hand, an RCT with a low risk of bias showed a higher pain reduction with superficial dry needling than with deep dry needling (MCID = 1.4 points) [29]. Our findings are also similar to those of a study that compared superficial dry needling and stretching exercises with stretching exercises only or no treatment [37]. However, to our knowledge, no other previous studies have compared superficial and deep dry needling in any musculoskeletal condition.

Regarding disability, our review also suggested that superficial or deep dry needling may be helpful in improving function. Nevertheless, only two RCTs with a high risk of bias [17,20] indicated that deep dry needling was superior to superficial dry needling in reducing disability, with reported improvements of 6 points for NDI and 12.26% for FRI. These reported disability reductions surpassed the previously established MCIDs for NDI (5 points) [43,44] and FRI (11–12%) [45,46]. Our findings are also similar to a couple of previous studies that found no significant difference between deep and superficial dry needling for functional disability in people with different levels of spinal pain [15,36].

The variability in the application of superficial and deep dry needling among the included studies, particularly in terms of needling application, depth of needle insertion and the length of the needle used, was quite significant. However, the depth of needle insertion was more consistent among the studies for deep dry needling than superficial dry needling. Thus, there was no consensus on the optimal treatment parameters, such as needling application, length of needles used, depth of needle insertion (particularly in the superficial dry needling procedure), number of treatment sessions and follow-up duration.

### Strengths and Limitations

A strength of the current systematic review is that it followed the PRISMA protocol and every step of the guidelines recommended previously [23]. Moreover, statistical analysis was performed and principles of best-evidence synthesis using effect size were used to determine the clinical significance of the results, taking into consideration that meta-analysis was not plausible in the current review. However, several limitations must be acknowledged. First, the search was restricted to English-language studies, meaning that relevant studies in other languages could have been missed. This restriction may have introduced language and publication bias, thereby reducing the comprehensiveness and generalisability of our findings. Second, a meta-analysis was not conducted due to the considerable heterogeneity of the included trials in terms of study populations, intervention protocols (depth, number of sessions, needle length), outcome measures, and follow-up durations. Although random-effects models are sometimes used to account for variability, the extent of clinical and methodological heterogeneity in this review was deemed too great to yield meaningful pooled estimates. For this reason, a narrative synthesis supported by effect size calculation was applied to provide a more transparent and clinically interpretable summary of the evidence. While this approach adds clarity, it also limits the overall strength of the conclusions. Future reviews with a larger and more homogeneous pool of trials may be able to perform meta-analyses to provide stronger and more generalisable estimates of effectiveness.

Finally, recent physiotherapy-specific effect-size guidance provides thresholds for small, medium and large group effects that can be used to estimate the minimum sample sizes required to detect clinically relevant effects [47]. Using these thresholds and conventional sample-size assumptions (two-sided α = 0.05, power = 80%), the approximate per-group sample sizes are ~25 for a large effect (d ≈ 0.8), ~99 for a medium effect (d ≈ 0.4) and ~1570 for a small effect (d ≈ 0.1). This indicates that the included RCTs were generally sufficiently powered to detect large effects and, in some cases, medium effects, but were too small to reliably detect small effect sizes. As a result, while the observed positive short-term outcomes are compatible with moderate-to-large treatment effects, the current literature lacks the statistical power to exclude or confirm small but potentially meaningful clinical effects. Future trials should therefore be designed with adequate sample sizes informed by these thresholds and include extended follow-up periods to assess sustainability of effects.

For the clinical implications of the current study’s findings, at least a single session of deep or superficial dry needling seems to be effective for short-term pain and functional disability reduction associated with MTrPs in patients with neck pain. However, these results should be interpreted with caution because most of the included RCTs were classified as having some risk-of-bias concerns or having a high risk of bias.

## 5. Conclusions

Our findings suggest that at least a single session of either deep or superficial dry needling will result in a short-term meaningful reduction in pain and functional disability associated with MTrPs in patients with neck pain. However, these findings should be treated with caution due to the heterogeneity of the treatment protocols and the high risk of bias in most of the included RCTs. For deep dry needling, we recommend the use of a 25–50 mm long and 0.25 mm diameter needle for 8–10 fast in-and-out movements. For superficial dry needling, it may be ideal to use a 5–15 mm long and 0.25 mm diameter needle for 8–10 fast in-and-out movements and then to keep it inserted for up to 15 min. These recommendations warrant further research to standardise the dry needling treatment protocol in terms of the number of sessions, treatment duration and application technique.

## Figures and Tables

**Table 1:** Inclusion and exclusion criteria.

PICO Element	Inclusion Criteria	Exclusion Criteria
Population (P)	Adults with neck pain and active myofascial trigger points (MTrPs) in the upper trapezius	Patients with radiculopathy Patients with whiplash injury Post-surgical patients Patients using analgesic medication Patients receiving other physiotherapy interventions
Intervention (I)	Deep dry needling	—
Comparator (C)	Superficial dry needling	—
Outcomes (O)	Pain intensity and functional disability	—
Other criteria	Study design: Randomized controlled trials (RCTs) Language: English only	Non-RCTs Non-English publications

**Table 2:** Inter-rater agreement between the reviewers for article screening.

Measure	Value
Total number of articles screened	127
Articles included by both reviewers	55 (43.3%)
Articles excluded by both reviewers	72 (56.7%)
Articles with disagreements	0 (0%)
Percentage of agreement	100%
Cohen’s kappa (κ)	1.00
95% confidence interval for kappa	1.00–1.00
Statistical significance (*p*-value)	<0.001

**Table 3:** Characteristics of the selected studies.

Authors	Sample Size	Diagnostic Criteria	Interventions	No. of Sessions	Follow-Ups	Outcome Measures	Main Results
Chys et al., 2023 [28]	54 (DDN: 26, SDN: 28)	Palpable tight band, local pain on pressure and referred pain	DDN vs. SDN in the upper trapezius	1 session	Immediately post-treatment	PPT CPM	There were no significant differences between DDN and SDN for PPT at local or distant sites. DDN significantly improved the relative CPM efficiency.
Ezzati et al., 2018 [17]	50 (DDN: 25, SDN: 25)	Palpable tight band, local pain on pressure and recognised pain	DDN vs. SDN in the upper trapezius	3 sessions	15 days	VAS NDI ROM	Both groups improved, but DDN showed greater gains in ROM and NDI over follow-up.
Hoseininejad et al., 2023 [18]	50 (DDN: 25, SDN: 25)	Neck/shoulder pain with at least one active trigger point in the upper trapezius persisting for 3 months	DDN vs. SDN in the upper trapezius	1 session	1 week	VAS NDI sEMG	Both groups improved in VAS and NDI, but only DDN significantly increased sEMG.
Martín-Rodríguez et al., 2019 [29]	34 (DDN: 17, control: 17)	Palpable tight band with local and familiar pain, and restricted ROM during full extension	Trigger point DDN vs. sham dry needling	1 session	1 month	CMC VAS ROM NDI	DDN improved pain, ROM and motor control, but there were no significant differences compared to sham DDN.
Myburgh et al., 2012 [30]	77 (symptomatic/asymptomatic)	Symptomatic group: significant MTrP and self-reported pain ≥ 3 on NRS-101. Asymptomatic group: no MTrP or pain (0).	DDN vs. SDN in the upper trapezius	1 session	28 h post-treatment	PPT NRS-101 F-max RFD	Both groups reduced pain, but PPT decreased across all participants. There were no significant differences in F-max or RFD.
Navarro et al., 2022 [31]	180 (DDN: 60, SDN: 60, placebo: 60)	Presence of latent MTrPs in the upper trapezius	DDN vs. SDN vs. placebo	1 session	1 week	PPT ACROM	Both DDN and SDN improved PPT and ROM over time, but DDN showed better ipsilateral rotation improvement at 7 days.
Sarrafzadeh et al., 2018 [19]	50 (DDN: 25, SDN: 25)	Palpable tight band, local pain on pressure and recognition of pain by the participants	DDN vs. SDN in the upper trapezius	3 sessions	15 days	VAS Ultrasonic evaluation	Both DDN and SDN reduced pain and increased muscle thickness, but DDN was superior in pain reduction.
Sedighi et al., 2017 [20]	30 (DDN: 15, SDN: 15)	Unilateral neck pain spreading to the frontotemporal area, worsened by movement, restricted ROM and C1–C3 tenderness	DDN vs. SDN in the suboccipital/upper trapezius	1 session	1 week	HI Pain intensity TrP tenderness ROM FRI	Both groups reduced HI and tenderness, but DDN showed superior improvements in ROM and FRI.

ACROM, active cervical range of motion; CMC, cervical motor control; CPM, conditioned pain modulation; DDN, deep dry needling; Fmax, maximum voluntary contraction; FRI, Function Rating Index; HI, headache index; NDI, Neck Disability Index; NRS-101, numerical rating scale; PPT, pain pressure threshold; RFD, rate of force development; ROM, range of motion; sEMG, surface electromyography; SDN, superficial dry needling; VAS, visual analogue scale.

**Table 4:** Inter-rater agreement between the reviewers for risk-of-bias assessment.

Domain	Percentage of Agreement (%)	Cohen’s Kappa (κ)	95% Confidence Interval (CI)	*p*-Value
Domain 1	87.5%	0.73	0.28–1.00	0.009
Domain 2	100%	1.00	1.00–1.00	0.005
Domain 3	100%	1.00	1.00–1.00	0.005
Domain 4	100%	1.00	1.00–1.00	0.000
Domain 5	87.5%	0.75	0.30–1.00	0.028
Overall	87.5%	0.78	0.646–1.00	0.002

**Table 5:** Risk of bias of the included studies using the Cochrane Risk of Bias 2 tool.

Study	Bias Due to the Randomisation Process	Bias Due to Deviation from the Intended Interventions	Bias Due to Missing Outcome Data	Bias in Outcome Measurement	Bias in the Selection of the Reported Result	Overall Risk of Bias
Chys et al., 2023 [28]	Low	Low	Low	Low	Low	Low
Ezzati et al., 2018 [17]	High	Low	Low	High	Some concerns	High
Hoseininejad et al., 2023 [18]	Some concerns	Low	Low	Low	Some concerns	Some concerns
Martín-Rodríguez et al., 2019 [29]	Low	Low	Low	Low	Low	Low
Navarro et al., 2022 [31]	Some concerns	Low	Low	Low	Low	Some concerns
Sarrafzadeh et al., 2018 [19]	Some concerns	Low	Low	Low	Low	Some concerns
Sedighi et al., 2017 [20]	Some concerns	High	Low	Some concerns	Some concerns	High

## Data Availability

The original contributions presented in this study are included in the article. Further inquiries can be directed to the corresponding author(s).

## Abbreviations

The following abbreviations are used in this manuscript:

MPS	Myofascial pain syndrome
MTrPs	Myofascial trigger points
RCTs	Randomised controlled trials
MCIDs	Minimal clinically important differences

## Appendix A. Search Strategy

**Database**	**Date of Search**	**Search Strategy**	**Results**
PubMed	22 September 2024	((“Neck Pain”[Mesh] OR “Cervical Pain”[Mesh]) AND (“Dry Needling”[Mesh] OR “deep dry needling” OR “Superficial dry needling”)) AND (“Trigger Points”[Mesh] OR “Myofascial Pain Syndromes”[Mesh])	22
Web of Science	22 September 2024	TS = (“Neck pain” OR “Cervical pain”) AND TS = (“deep dry needling” OR “Superficial dry needling”) AND TS = (“Trigger point*” OR “Myofascial pain syndrome”)	20
Scopus	22 September 2024	(TITLE-ABS-KEY (“Neck pain” OR “Cervical pain”)) AND (TITLE-ABS-KEY (“deep dry needling” OR “Superficial dry needling”)) AND (TITLE-ABS-KEY (“Trigger point*” OR “Myofascial pain syndrome”))	12
Embase	22 September 2024	(‘neck pain’ OR ‘cervical pain’:ab,ti,kw) AND (‘deep dry needling’ OR ‘superficial dry needling’:ab,ti,kw) AND (‘trigger point*’ OR ‘myofascial pain syndrome’:ab,ti,kw)	14
Google Scholar	22 September 2024	intitle:(“Neck pain” OR “Cervical pain”) AND intitle:(“deep dry needling” OR “Superficial dry needling”) AND intitle:(“Trigger point*” OR “Myofascial pain syndrome”)	51
Dimensions	22 September 2024	(“Neck pain” OR “Cervical pain”) AND (“deep dry needling” OR “Superficial dry needling”) AND (“Trigger point*” OR “Myofascial pain syndrome”)	10
OpenAlex	22 September 2024	(“Neck pain” OR “Cervical pain”) AND (“deep dry needling” OR “Superficial dry needling”) AND (“Trigger point*” OR “Myofascial pain syndrome”)	63
